# Photo-Induced Oxidative Stress Impairs Mitochondrial Metabolism in Neurons and Astrocytes

**DOI:** 10.1007/s12035-017-0720-2

**Published:** 2017-08-24

**Authors:** Elena Berezhnaya, Maria Neginskaya, Anatoly B. Uzdensky, Andrey Y. Abramov

**Affiliations:** 1 Laboratory of Molecular Neurobiology, Academy of Biology and Biotechnology, Southern Federal University, pr. Stachki 194/1, Rostov-on-Don, 344090 Russia; 2 Department of Molecular Neuroscience, UCL Institute of Neurology, Queen Square, London, WC1N 3BG UK

**Keywords:** Photodynamic therapy, Radachlorin, Neurons, Astrocytes, Mitochondrial potential, NADH, Poly (ADP-ribose) polymerase (PARP)

## Abstract

Photodynamic therapy is selective destruction of cells stained with a photosensitizer upon irradiation with light at a specific wavelength in the presence of oxygen. Cell death upon photodynamic treatment is known to occur mainly due to free radical production and subsequent development of oxidative stress. During photodynamic therapy of brain tumors, healthy cells are also damaged; considering this, it is important to investigate the effect of the treatment on normal neurons and glia. We employed live-cell imaging technique to investigate the cellular mechanism of photodynamic action of radachlorin (200 nM) on neurons and astrocytes in primary rat cell culture. We found that the photodynamic effect of radachlorin increases production of reactive oxygen species measured by dihydroethidium and significantly decrease mitochondrial membrane potential. Mitochondrial depolarization was independent of opening of mitochondrial permeability transition pore and was insensitive to blocker of this pore cyclosporine A. However, irradiation of cells with radachlorin dramatically decreased NADH autofluorescence and also reduced mitochondrial NADH pool suggesting inhibition of mitochondrial respiration by limitation of substrate. This effect could be prevented by inhibition of poly (ADP-ribose) polymerase (PARP) with DPQ. Thus, irradiation of neurons and astrocytes in the presence of radachlorin leads to activation of PARP and decrease in NADH that leads to mitochondrial dysfunction.

## Introduction

Photodynamic therapy is selective destruction of cells stained with a photosensitizing agent (photosensitizer) upon irradiation with light at a specific wavelength in the presence of oxygen [1, 2]. It is applied in oncology for treatment of different tumors, including brain tumors [3, 4]. Cell death upon photodynamic treatment is known to occur mainly due to production of reactive oxygen species (ROS) by the photosensitizer [1]. The exact mechanisms of the cell death could vary for different photosensitizing agents depending on the places of their accumulation, their ability to produce ROS, etc. [5]. One of the promising photosensitizers undergoing preclinical trials in Netherlands toward achieving clinical acceptance in the European Union is Radachlorin®, also known as Bremachlorin® [6, 7]. During photodynamic therapy of brain tumors, healthy cells are also damaged; considering this, unraveling the effects of irradiation of photosensitizers on normal neurons and glial cells is important for prevention of possible side effects during the treatment.

Brain cells consume 10 times more glucose and oxygen than other tissues, and any changes in energy metabolism induce energy deprivation in some pathologies followed by cell death [8]. Main cellular energy producer—mitochondria—is also involved in the mechanism of the cell death. Although a mitochondrion is one of the major producers of ROS in the cells, it is also one of the major targets of oxidative damage [9]. Oxidative stress in mitochondria results in oxidation of the proteins of the respiratory complexes, lipid peroxidation, and DNA damage.

One of the DNA-repairing enzymes—poly (ADP-ribose) polymerase (PARP)—plays an important role in the protection of the genetic information in the cells. However, prolong and intensive activation of the PARP can lead to pathology due to toxicity of the product of this enzyme PAR or energy deprivation due to consumption of NAD^+^ and decrease of NADH for mitochondrial electron transport chain [10, 11]. Activation of PARP is shown to be involved in the number of brain pathologies: Alzheimer’s disease [11, 12], Parkinson’s disease [13, 14], and glutamate toxicity [15, 16].

In this study, we unraveled cellular mechanisms of photodynamic action of Radachlorin® on mitochondria of primary co-culture of cortical neurons and astrocytes. We have found that illumination of cells with the radachlorin leads to mitochondrial depolarization in both neurons and astrocytes and decrease of mitochondrial NADH level.

## Materials and Methods

### Primary Co-culture Preparation

Mixed primary co-cultures of hippocampal, cortical neurons, and astrocytes were prepared as described previously [17]. Hippocampi and cortices were removed into ice-cold PBS (Ca^2+^, Mg^2+^-free, Invitrogen, Paisley, UK). The tissue was minced and trypsinized (0.25% for 5 min at 37 °C), triturated and plated on poly-D-lysine-coated coverslips, and cultured in Neurobasal A medium (Invitrogen, Paisley, UK) supplemented with B-27 (Invitrogen, Paisley, UK) and 2 mM L-glutamine. Experimental procedures were performed in full compliance with the United Kingdom Animal (Scientific Procedures) Act of 1986. Cultures were maintained at 37 °C in a humidified atmosphere of 5% CO_2_ and 95% air, media changed twice a week. Cells were used at 10–15 days in vitro unless stated differently. Neurons were easily distinguishable from glia: they appeared phase bright, had smooth rounded somata, and distinct processes, and lay just above the focal plane of the glial layer.

### Photodynamic Treatment

To induce photodynamic damage of cells, we used the photosensitizer Radachlorin®, which is the mixture of the sodium salts of chlorine e6, chlorine p6, and purpurin 18 [7]. Photodynamic treatment varied depending on the type of the experiment. Radachlorin concentration was chosen empirically according to our previous data on neuron firing and cell death in crayfish stretch receptor [7] and calcium response that was observed during PDT in the same culture of neurons and astrocytes and described in details in [18]. PDT with 200 nm radachlorin corresponds to the treatment, at which calcium signal is induced and ROS production is already elevated, but cells do not die simultaneously. Cells were preincubated with radachlorin at this concentration for 5–10 min or it was added during the experiment. Then, the culture was irradiated with a diode laser at 0.1 mW/cm^2^ and 654 nm. The duration of exposure in different experiments varied from 30 s to 5 min.

### Live-Cell Imaging

Fluorescence measurements were obtained on an epifluorescence-inverted microscope equipped with a 20× fluorite objective. Emitted fluorescence light was reflected through a filter to a cooled CCD camera (Retiga, QImaging, Canada) and digitized to 12-bit resolution. All imaging data were collected using software from Andor (Belfast, UK) and analyzed using ImageJ.

For measurements of mitochondrial membrane potential (∆Ψ_m_), cells were loaded for 10 min at room temperature with 1 μM Rhodamine 123 (Rh123; Molecular Probes, Eugene, OR) in HEPES buffered salt solution (HBSS) composed of (mM): 156 NaCl, 3 KCl, 2MgSO_4_, 1.25 KH_2_PO_4_, 2 CaCl_2_, 10 glucose, and 10 HEPES, pH adjusted to 7.35. For measurement of ROS production, dihydroethidium (HEt; 2 μM; Molecular Probes, Eugene, OR) was used.

Mitochondrial potential (ΔΨ_m_) was measured in single cells using excitation light provided by a Xenon arc lamp, the beam passing through a monochromator at 490 nm with emission above 510 nm. For HEt measurements, the excitation wavelength was 360 nm and emission wavelength was 430 nm for non-oxidized DHE, and excitation 530 nm and emission 560 nm for oxidized HEt. Accumulation of Rh123 in polarized mitochondria quenches the fluorescent signal, and in response emission is dequenched; an increase in Rh123 signal therefore indicates mitochondrial depolarization.

NAD(P)H autofluorescence was measured in cells plated on 2-mm glass coverslips using an epifluorescence-inverted microscope equipped with a 20× fluorite objective. For measurement of NAD(P)H in single cells, excitation light at a wavelength of 350 nm was provided by a Xenon arc lamp, the beam passing through a monochromator (Cairn Research, Faversham, Kent, UK). Emitted fluorescence light was reflected through a 455-nm long-pass filter to a cooled CCD camera (Retiga, QImaging, Surrey, BC, Canada) and digitized to 12-bit resolution. All imaging data were collected using software from Andor (Belfast, UK) and analyzed using ImageJ.

### Statistical Analysis

Results for ROS and mitochondrial potential are expressed as means ± the standard error of the mean (SEM); repeated measures ANOVA and Student’s *t* test were used, respectively. Statistical analysis was performed using Origin 8.1 (Microcal Software Inc., Northampton, MA, USA) software.

## Results

### Photodynamic Treatment with Radachlorin Induces ROS Production

**Fig. 1:**
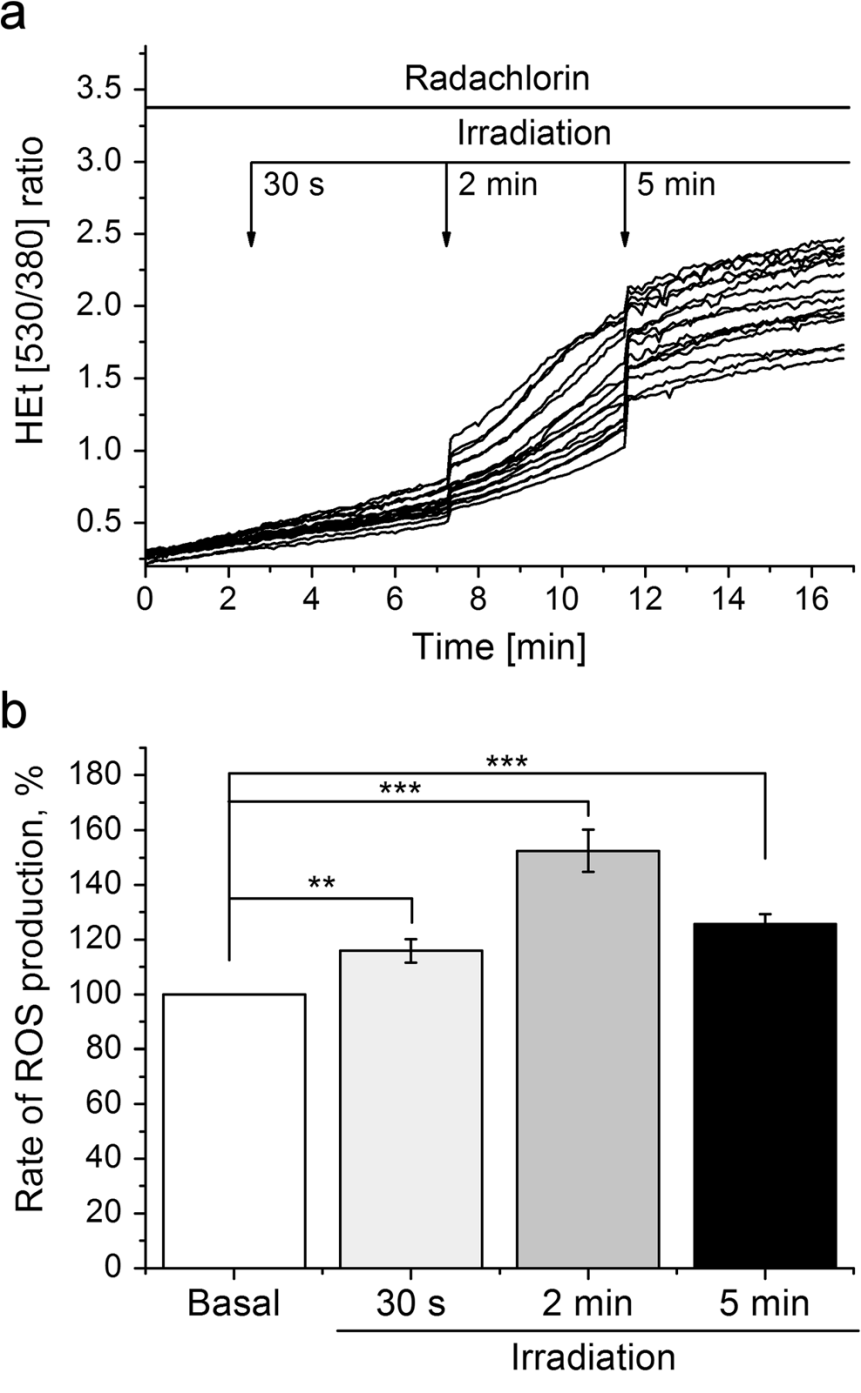
The photodynamic effect of radachlorin (200 nM) induces production of reactive oxygen species (ROS) in primary co-culture of neurons and astrocytes. The effect depends on the duration of irradiation. It is the most pronounced after irradiation for 2 min compared to 10 s and 5 min. **a** shows representative traces of dihydroethidium (HEt) measurements, whereas **b** summarizes the effects of irradiations for 30 s, 2 min, and 5 min on the ROS production in percentage. **p < 0.01, ***p < 0.001 as compared with the basal level

Incubation of the rat cortical primary co-cultures of neurons and astrocytes with 200 nm radachlorin did not change the rate of ROS production measured as a rate of oxidation of dihydroethidium (Fig. 1 a). Irradiation of the cells for 30 s, 2 min, and 5 min induced an increase in the rate of ROS production (116 ± 4, *n* = 164 cells, *p* < 0.01; 153 ± 8, *n* = 164 cells, *p* < 0.001, and 126 ± 4, *p* < 0.001; of basal rate 100%, *n* = 164 cells; accordingly, Fig. 1b). This effect depended on the duration of irradiation with the most pronounced increase of the ROS after irradiation for 2 min compared to 30 s and 5 min (*p* < 0.5, Fig. 1b). Thus, irradiation of the cells with the radachlorin induces ROS production in the cortical co-culture of neuron and astrocytes.

### Photodynamic Treatment with Radachlorin Induces Mitochondrial Depolarization

**Fig. 2:**
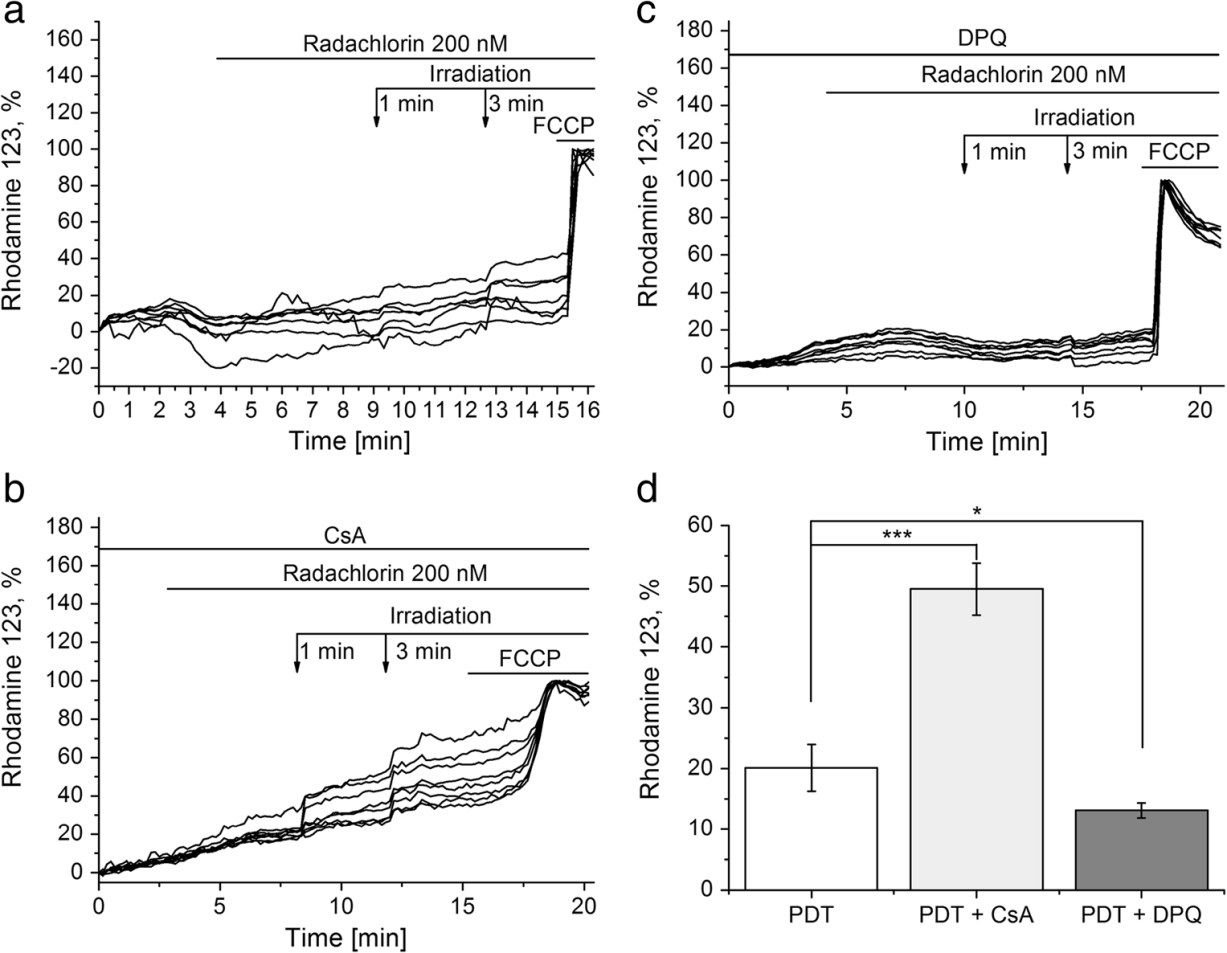
Irradiation of the Radachlorin loaded rat primary co-culture of neurons and astrocytes decreases mitochondrial membrane potential (ΔΨm). Changes in ΔΨm with time were measured using Rhodamine 123 in “dequench” mode (where the loss of potential is seen as an increase in fluorescence). **a** Radachlorin (200 nM) itself causes mitochondrial hyperpolarization, while irradiation in its presence leads to mitochondrial depolarization. **b** Cyclosporin A (CsA, 1 μM) inhibits the effect of radachlorin in the dark, but not when irradiated. **c** A PARP inhibitor, DPQ (20 μM), partially blocked both effects. **d** The level of mitochondrial depolarization after PDT and before addition of FCCP in percentage in the absence (*n* = 8) and presence of the inhibitors CsA (*n* = 8) and DPQ (*n* = 14). *p < 0.5, ***, *p* < 0.001 as compared with irradiation in the absence of the inhibitors

Mitochondrial membrane potential (∆Ψ_m_) is the major indicator of mitochondrial health. Application of 200 nM radachlorin to rat primary co-culture of neurons and astrocytes induced mitochondrial hyperpolarization, suggesting that radachlorin by itself does not inhibit mitochondrial metabolism. However, irradiation of the cells loaded with radachlorin for 1 and 3 min resulted in a significant loss of ∆Ψ_m_ (by 20 ± 4%, *n* = 8, Fig. 2a, d). ROS is one of the major triggers for opening the mitochondrial permeability transition pore (mPTP; for review see [19]) that can induce changes in ∆Ψ_m_. An inhibitor of mPTP, Cyclosporin A (CsA, 1 μM), had no effect on mitochondrial depolarization induced by irradiation of radachlorin (*n* = 8, Fig. 2b, d). Moreover, incubation of cells with CsA enhanced the effect of cell irradiation on the mitochondrial membrane potential (50 ± 4%, *n* = 8, Fig. 2b, d). ROS can induce the DNA oxidation and stimulate of the DNA-repairing mechanisms. Incubation of the cells with PARP inhibitor, DPQ (20 μM, 20 min), significantly reduced mitochondrial depolarization induced by radachlorin irradiation (13 ± 1%, *n* = 14, *p* < 0.05, Fig. 2c, d).

### Photodynamic Treatment with Radachlorin Decreases NADH Autofluorescence

There are several possible explanations for a reduction in ΔΨ_m_, from direct damage of the proteins of mitochondrial respiratory chain to mitochondrial uncoupling or inhibition of the substrate delivery to electron transport chain. In order to investigate mitochondrial respiration specifically in either neurons or astrocytes, we measured NAD(P)H autofluorescence in co-cultures of primary neurons and astrocytes and calculated the redox index. NADH is the electron donor for complex I, and as such NADH levels correlate inversely with respiratory chain activity or to the rate of production of NADH in TCA cycle. In order to separate mitochondrial NADH autofluorescence from cytosolic NADH and cellular NADPH signal, we added FCCP (1 μM) to minimize the mitochondrial NADH pool (due to maximized respiration) followed by application NaCN (1 mM) to maximize the NADH pool (by blocking mitochondrial respiration and consumption of NADH in complex I) [20]. The total mitochondrial pool of NADH (maximum autofluorescence minus minimum) may be taken as an indication of the substrate availability for complex I.

**Fig. 3:**
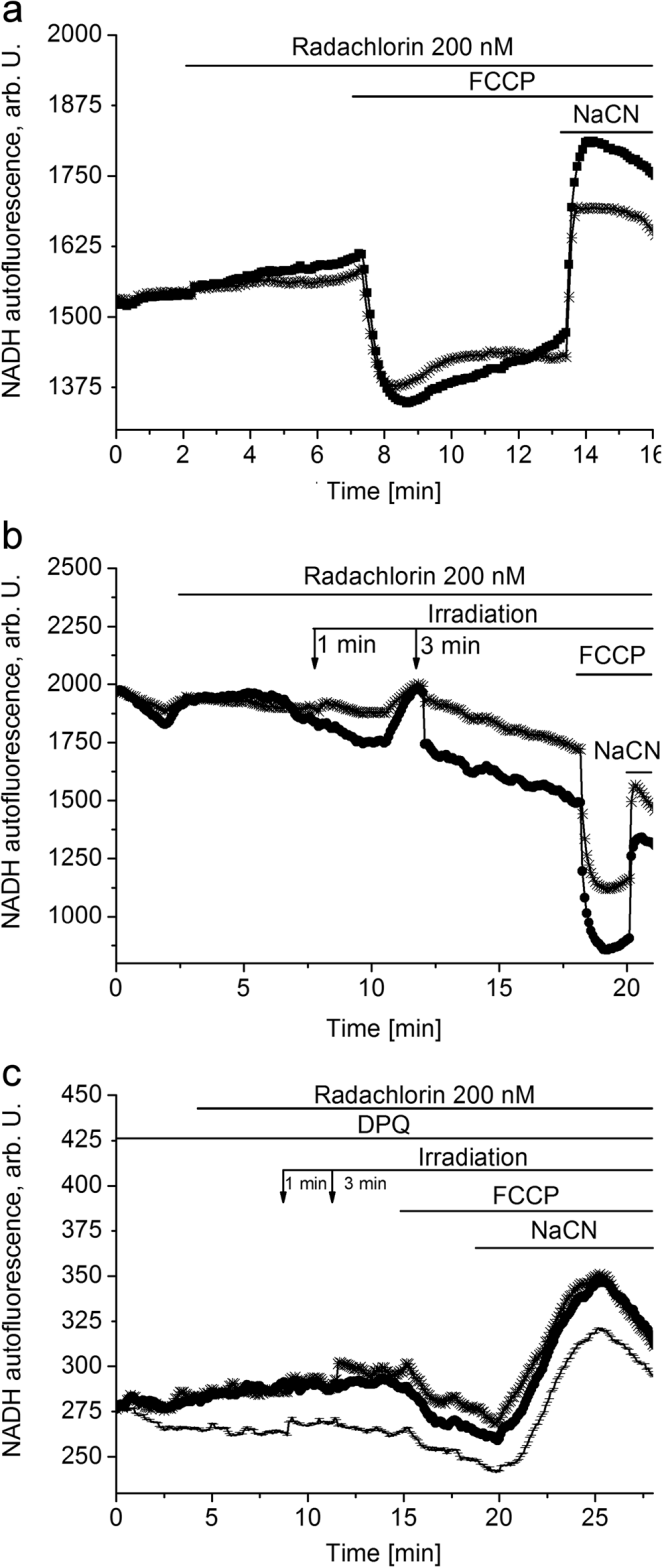
Radachlorin does not affect NADH autofluorescence, while irradiation (1 and 3 min) in its presence decreases it. **a** Application of 200 nM radachlorin in the dark did not affect the NADH autofluorescence signal in primary co-culture of neurons and astrocytes. **b** Irradiation (1 and 3 min) in the presence of radachlorin (200 nM) caused a decrease in the NADH autofluorescence signal and reduced the mitochondrial NADH pool compared to cells that were not irradiated. **c** A PARP inhibitor, DPQ (20 μM), inhibited a decrease in the NADH autofluorescence that was induced by irradiation in the presence of radachlorin

Application of radachlorin (200 nM) induced no visual changes in mitochondrial NADH autofluorescence (*n* = 53, Fig. 3a), while further irradiation (1 and 3 min) decreased it (*n* = 44, Fig. 3b). It should be noted that the effect of NaCN was significantly lower than the basal level (Fig. 3b) that suggests the inhibition of the NADH production and/or reduction of the mitochondrial NADH pool (Fig. 3b). Importantly, a PARP inhibitor, DPQ (20 μM), blocked a decrease in the NADH autofluorescence induced by the photodynamic treatment with radachlorin, and the effect of NaCN in the end of the experiment was significantly higher that strongly suggests that a decrease in the mitochondrial NADH pool is induced by lower production of NADH due to the limitation of NAD^+^ (*n* = 6, Fig. 3c).

## Discussion

PDT with radachlorin is effective for destruction of tumor cells [21]. It leads to free radical production with a more pronounced increase after 2 min irradiation, when compared to 5 min (*p* < 0.5, Fig. 1b). It could originate from ROS-producing enzymes in the cells [22], whereas further irradiation for 5 min could be damaging for them and, thus, a lower level of ROS is observed. Here we also show that PDT with radachlorin alters mitochondrial metabolism in neurons and astrocytes that can be a possible mechanism for cell death as for targeted cells as well as for surrounding healthy cells. A change of the mitochondrial metabolism in brain tissue is of special importance considering high energy demand in neurons [8].

Involvement of PARP activation and energy deprivation in neuronal death was shown for a number of pathologies including excitotoxicity and neurodegenerative disorders [11, 15, 16]. Mitochondria are a key player in the mechanism of the cell death [23]. Release of pro-apoptotic proteins from mitochondria is triggered by opening of the mitochondrial permeability transition pore (mPTP). Free radical production and lower mitochondrial membrane potential are triggers for induction of mPTP [19]. PDT with radachlorin (but not radachlorin alone) produces ROS and can reduce mitochondrial membrane potential. Although we did not observe mPTP opening during irradiation of cells with radachlorin for short time (Fig. 2), we can suggest triggering this process after more prolonged PDT.

It is known that irradiation itself can lead to fading of NADH autofluorescence, which is observed in our experiments. A small increase in the NADH autofluorescence following addition of NaCN could be another evidence of this. But since it is blocked upon addition of PARP inhibitor, DPQ (20 μM), we suggest that it is rather activation of PARP than fading, which causes a decrease in the NADH autofluorescence.

NAD^+^ and energy deprivation that result from PARP activation are known to induce seizures [24]. That is why this effect of PDT with radachlorin on healthy neurons and glial cells should be taken into account upon treatment of brain tumors. ROS-induced energy deprivation may not produce damage and initiation of the cell death for resting cells. However, in energy-demanding processes, such activation of the calcium signaling by glutamate release of epilepsy-like activity, this lack of ATP may induce cell death [25, 26].
